# Multiple hydrogen-bonded dimers: are only the frontier atoms relevant?†

**DOI:** 10.1039/d3cp05244c

**Published:** 2023-11-30

**Authors:** Celine Nieuwland, David Almacellas, Mac M. Veldhuizen, Lucas de Azevedo Santos, Jordi Poater, Célia Fonseca Guerra

**Affiliations:** a Department of Chemistry and Pharmaceutical Sciences, Amsterdam Institute for Molecular and Life Sciences (AIMMS), Vrije Universiteit Amsterdam De Boelelaan 1108 Amsterdam 1081 HZ The Netherlands c.fonsecaguerra@vu.nl https://www.theochem.nl/; b Departament de Química Inorgànica i Orgànica & Institut de Química Teòrica i Computacional, Universitat de Barcelona Martí i Franquès 1-11 Barcelona 08028 Catalonia Spain; c ICREA Pg. Lluís Companys 23 Barcelona 08010 Spain

## Abstract

Non-frontier atom exchanges in hydrogen-bonded aromatic dimers can induce significant interaction energy changes (up to 6.5 kcal mol^−1^). Our quantum-chemical analyses reveal that the relative hydrogen-bond strengths of *N*-edited guanine–cytosine base pair isosteres, which cannot be explained from the frontier atoms, follow from the charge accumulation in the monomers.

## Abstract 2

Non-frontier atom exchanges in hydrogen-bonded aromatic dimers can induce significant interaction energy changes (up to 6.5 kcal mol^−1^), which can only be explained from the charge accumulation in the monomers.

## Graphical abstract

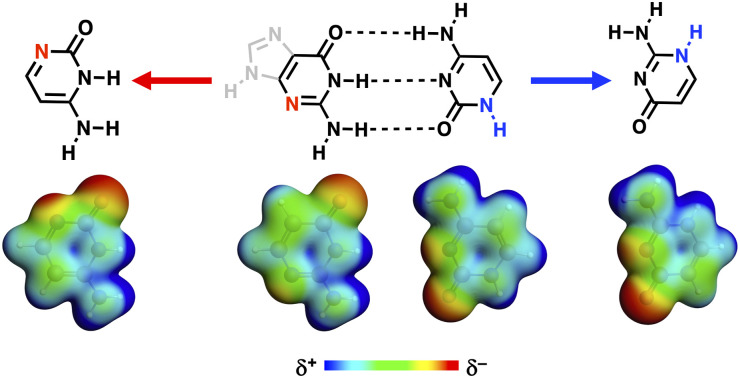

## Introduction

Intermolecular hydrogen bonding is one of the central interactions underlying self-assembly and molecular recognition in biochemistry, such as in protein folding and DNA duplex formation by constituting the hydrogen bonds (H-bonds) between the complementary DNA base pairs.^1^ The ability to self-assemble has inspired the field of supramolecular chemistry to incorporate intermolecular H-bonding in the design of novel catalysts,^2^ (macro)molecules,^3^ and materials.^4^ However, in order to rationally design new and improved molecules and materials, a profound understanding of the mechanism of intermolecular H-bonding and the prediction of the interaction strength is required.

The strength of an H-bond is often explained from the properties of the interacting frontier atoms, that is, the partially positively charged H-bond donor (*i.e.*, H(–O) or H(–N)) and the partially negatively charged H-bond acceptor (*i.e.*, O or N) groups.^5–7^ An example of this is the secondary electrostatic interaction (SEI) model^6^ from Jorgensen and Pranata which is widely used to predict and explain trends in intermolecular H-bond strengths.^7^ This model is based on the assumption that the H-bond strength between monomers can be predicted from the position and charge of the frontier atoms. However, the SEI model is physically incorrect due to the oversimplification of the H-bonding mechanism by regarding the H-bond donor and acceptor groups as interacting point charges. Popelier and Joubert^8^ showed that the consideration of only frontier-atom electrostatic interactions, like in the SEI model, is elusive because also the electrostatic interactions between distant atoms contribute to the stability of H-bonded pairs. More importantly, it is nowadays well-established that H-bonds are not purely electrostatic in nature but also contain a significant covalent character,^9^ which arises from the donation of electronic density from the filled σ-lone pair orbital of the H-bond acceptor into the empty antibonding σ* orbital on the H-bond donor. This additional stabilizing orbital-interaction component is in fact essential to overcome the destabilizing Pauli repulsion associated with H-bond formation (see ref. 9a for an overview of all the relevant interaction components of H-bonds). Our previous work into the predictive nature of the SEI model revealed that the relative stability of H-bonded pairs follows from measuring the charge accumulation in the monomers rather than from the secondary interactions between H-bond frontier atoms.^10,11^

**Fig. 1:**
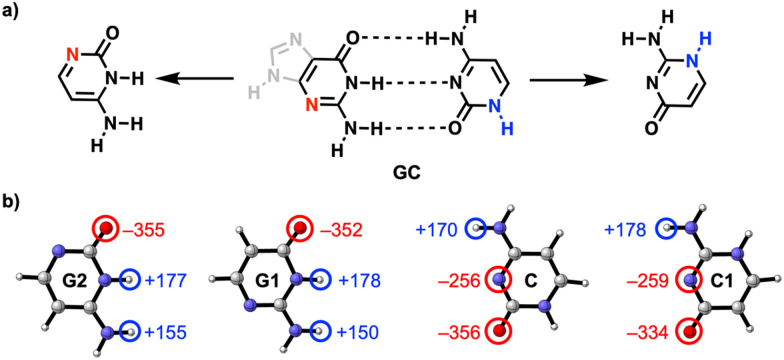
(a) Schematic structure of the guanine–cytosine (GC) base pair and *N*-edited isosteres used in this work to tune the intermolecular H-bond strength by the position of the non-frontier N and NH groups highlighted in red and blue, respectively. (b) Voronoi deformation density (VDD) atomic charges *Q* (in milli-electrons) of the H-bond frontier atoms of the isolated guanine (G1 and G2) and cytosine (C and C1) isosteres in the geometry within the dimer with C or G1, respectively.

In this work, we challenge the concept of explaining H-bond strengths by the frontier atoms further. We show that the binding strength of H-bonded pairs with identical H-bond frontier atoms can be adjusted by minimal non-frontier atom exchanges. Our dispersion-corrected density functional theory (DFT-D) based analyses at the ZORA^12^-BLYP^13^-D3(BJ)^14^/TZ2P^15^ level using the Amsterdam Modeling Suite^16^ (AMS2022.101) (see ESI,† Method S1 for the full computational details) show that the stability of mimics of the guanine–cytosine (GC) DNA base pair can be systematically tuned by varying the position of the non-frontier N and NH groups (see Fig. 1a). We demonstrate that these minimal non-frontier modifications have significant implications on the H-bond strength of the corresponding base pairs although the charges on the frontier atoms are barely affected in these so-called *N*-edited G and C isosteres (Fig. 1b). We find that the relative binding strengths can be rather explained and predicted by measuring the amount of charge accumulation in the monomers. This allows for control of the H-bond strength of these *N*-edited nucleobases, which can be applied in the design of innovative (bio)supramolecular building blocks.^17^

## Results and discussion

**Fig. 2:**
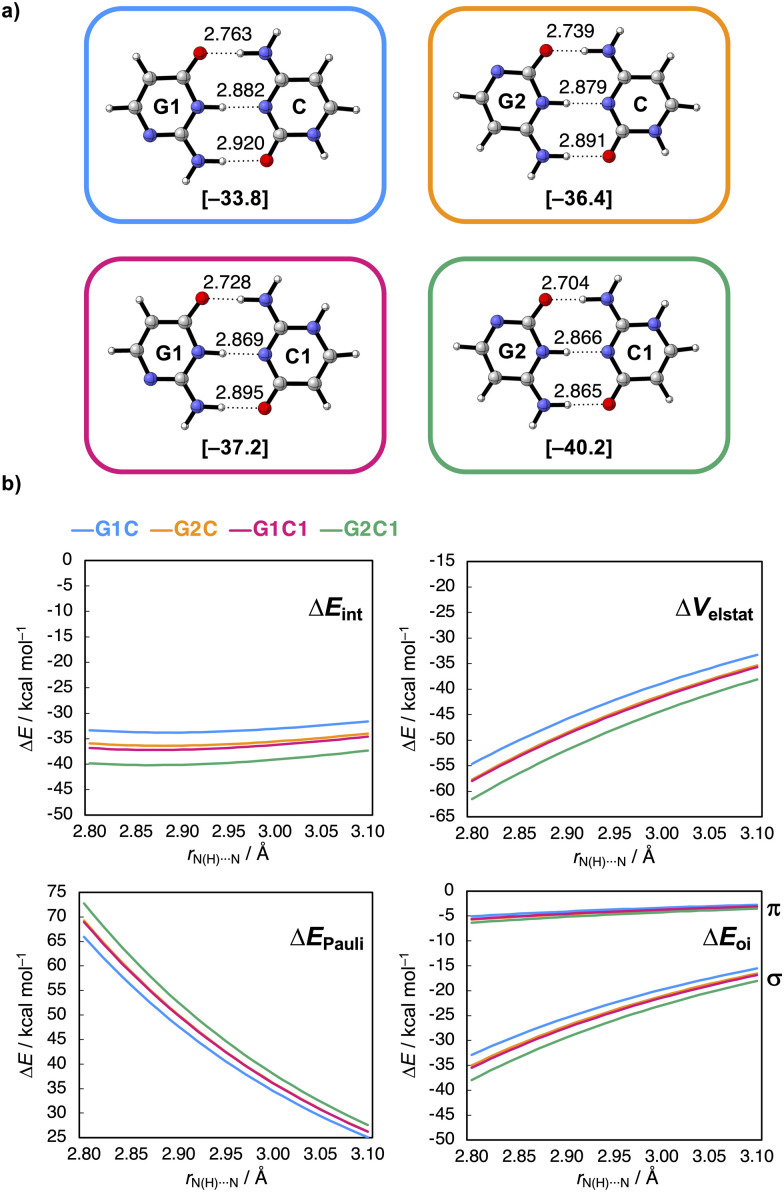
(a) Hydrogen-bonded GC base-pair isosteres with equilibrium hydrogen-bond distances (in Å) and interaction energy Δ*E*_int_ (in kcal mol^−1^ in between brackets) and (b) decomposed interaction energy terms (Δ*E*_int_ = Δ*V*_elstat_ + Δ*E*_Pauli_ + Δ*E*_oi_ (+ Δ*E*_disp_)) as a function of the middle H-bond distance *r*_N(H)⋯N_ (in Å, step size of 0.01 Å).

Based on the similar charges on the H-bond frontier atoms, shown in Fig. 1b, one would expect no significant change in the H-bond strength of the GC base pair upon changing the position of the non-frontier heteroatoms in the G and C monomers.^18^ However, we find that the interaction energy (Δ*E*_int_) of the GC H-bonded base pair analogues, presented in Fig. 2a, becomes more stabilizing from G1C (blue) < G2C (orange) < G1C1 (pink) < G2C1 (green), associated with a maximum stabilization of the H-bond interaction energy by *ca.* 6.5 kcal mol^−1^. So, the H-bond interaction is enhanced upon (i) changing the G1 isostere to G2, thereby grouping the partially negative N and O atoms on one side of the molecule, and (ii) upon changing C to C1, in which the partially positive NH_(2)_ groups are in closer proximity. Note that we discuss here the interaction energies because they dictate the trend in the relative H-bond strengths (see ESI,† Data S1 and see Method S2 for computational details).

To understand what causes this stabilization, Δ*E*_int_ was partitioned into four physically meaningful terms using a quantitative energy decomposition analysis (EDA):^19^ (i) the classical electrostatic interaction (Δ*V*_elstat_), (ii) the steric Pauli repulsion (Δ*E*_Pauli_) arising from the repulsion between overlapping closed-shell orbitals on the interacting monomers, (iii) the orbital interaction (Δ*E*_oi_) which accounts for charge transfer (*i.e.*, covalency) in the σ-electronic system and polarization of the π-electronic system, and (iv) the dispersion energy (Δ*E*_disp_) (see ESI,† Method S2 for a theoretical overview of this method).

The EDA can be performed on the equilibrium dimers (see ESI,† Data S1). However, as the H-bond distances vary for the different GC base-pair isosteres (see Fig. 2a), a more insightful picture can be obtained when performed at similar H-bond distances. This allows us to differentiate between interaction terms that are intrinsically more stabilizing, from the interaction terms that are simply enhanced by shortened H-bond distances. To this end, the EDA was performed as a function of the middle H-bond distance *r*_N(H)⋯N_ using the PyFrag 2019 software,^20^ in which the two monomers in the geometry of the equilibrium dimer approach each other as two frozen blocks (ESI,† Method S2 for details). The results of this analysis are presented in Fig. 2b and show that the stabilization of Δ*E*_int_ from G1C (blue) < G2C (orange) < G1C1 (pink) < G2C1 (green) is preserved along the entire H-bond distance range. Furthermore, it shows that Δ*E*_int_ becomes more stabilizing upon interchanging G1 to G2 and C to C1 because both the electrostatic (Δ*V*_elstat_) and orbital interactions (Δ*E*_oi_) become more stabilizing along this trend. For Δ*E*_oi_, in particular, the σ-charge transfer orbital interactions (Δ*E*^σ^_oi_) are enhanced, while the π-orbital interactions (Δ*E*^π^_oi_) do not really vary for the different isosteres. This means that the π-resonance assistance in the H-bonds, that is π-polarization, is not affected by the non-frontier modifications. Note that Δ*E*_Pauli_ does not dictate the trend, as this interaction component becomes more destabilizing from G1 to G2 and from C to C1 (Δ*E*_disp_ stays constant for all isosteres, see ESI,† Data S2).

**Fig. 3:**
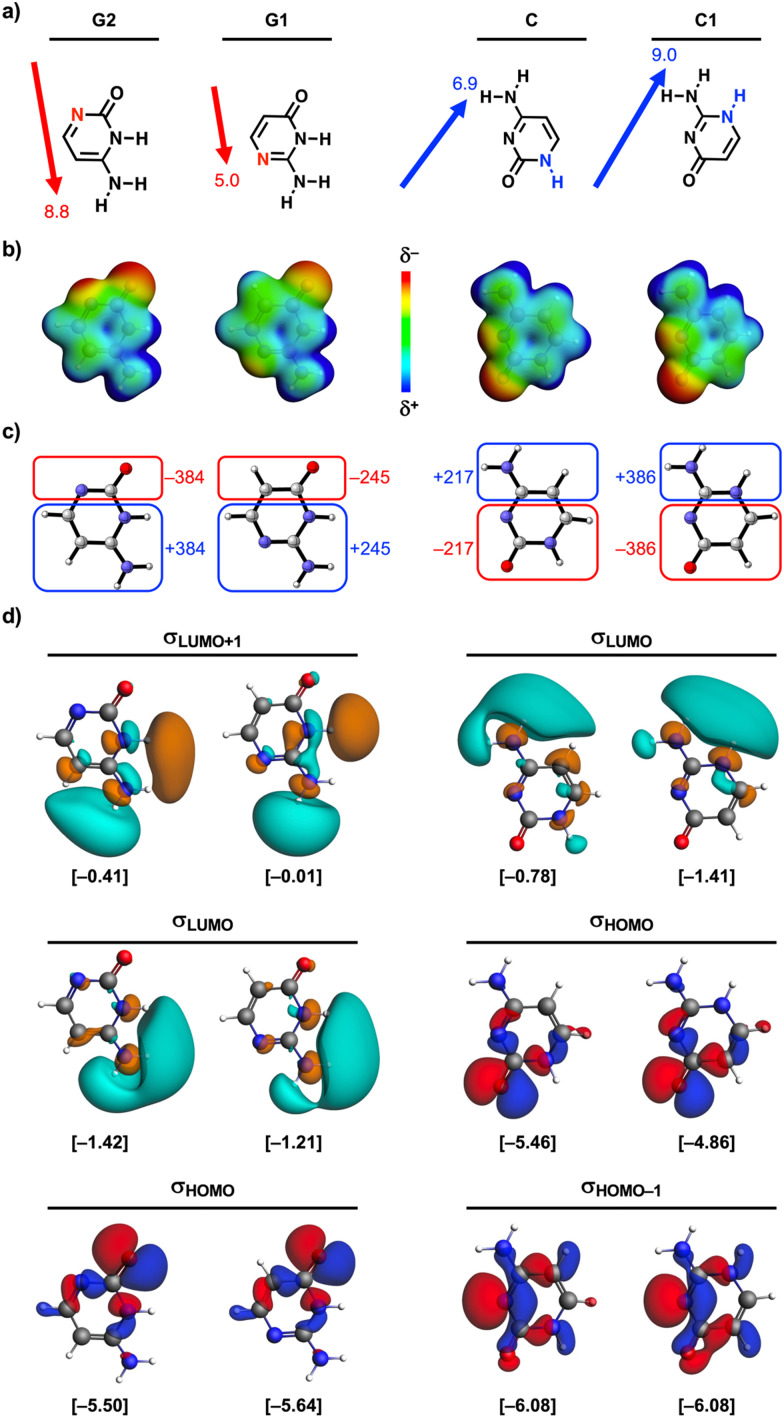
(a) Molecular dipole moment vectors (*δ*^−^ → *δ*^+^ direction) and magnitudes (in Debye), (b) molecular electrostatic potential surfaces (at 0.01 a.u.) from −0.1 (red) to 0.1 (blue) a.u., (c) Voronoi deformation density (VDD) atomic charges *Q* (in milli-electrons), and (d) isosurfaces (at 0.03 a.u.) with corresponding energies *ε* (in between brackets in eV) of the relevant H-bonding unoccupied (*σ*_LUMO_) and occupied (*σ*_HOMO_) orbitals of the isolated guanine (G1 and G2) and cytosine (C and C1) isosteres in the geometry within the dimer with C or G1, respectively.

While the similar frontier atomic charges of the isosteres cannot explain the enhancement of Δ*V*_elstat_ and Δ*E*^σ^_oi_ (*vide supra*), we find that the origin of this effect lies in the amount of charge accumulation in the *N*-edited monomers, as is presented in Fig. 3. Upon going from G1 to G2 and from C to C1, the partially negative (O and N) and partially positive (NH and NH_2_) functional groups, respectively, are grouped on one side of the molecule, which induces an increase of the molecular dipole moment (Fig. 3a). This grouping of the polar functional groups is associated with an increase of charge accumulation on both sides of the molecule, as is visualized by the electrostatic potential surfaces in Fig. 3b and the Voronoi deformation density (VDD)^21^ charges in Fig. 3c (see ESI,† Method S3 for details about the VDD method and Data S3 for the complete VDD charge analysis). Although the charge on the H-bond frontier atoms does not significantly change (Fig. 1b), the grouping of all polar groups on one side of the molecule by going from G1 to G2 and from C to C1, enhances the molecular charge accumulation, that is, the interacting monomers become more pronounced ‘mirrored dipoles’ of each other (see Fig. 3c), which enhances the intermolecular electrostatic interactions. This is a manifestation of the fact that the total electrostatic interaction is not exclusively determined by the frontier atoms but also by long-range electrostatic interactions between distant atoms.

**Fig. 4:**
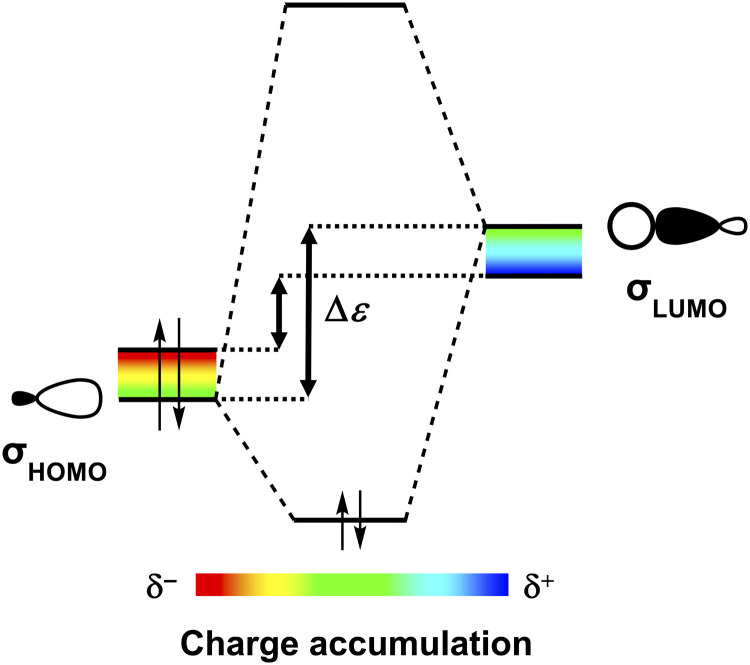
A larger accumulation of negative charge (*δ*^−^) destabilizes the occupied molecular orbitals (*σ*_HOMOs_) of H-bond acceptors, while a larger accumulation of positive charge (*δ*^+^) stabilizes the unoccupied molecular orbitals (*σ*_LUMOs_) of H-bond donors. This results in a smaller HOMO–LUMO energy gap (Δ*ε*) and therefore a better σ-orbital interaction in case of enhanced charge accumulation.

Besides Δ*V*_elstat_, the σ-orbital interactions Δ*E*^σ^_oi_ are also enhanced upon grouping the polar functional groups on one side of the nucleobases thereby increasing the molecular charge accumulation. A larger accumulation of negative charge destabilizes occupied orbitals (*i.e.*, they become better electron donors), while a larger accumulation of positive charge stabilizes unoccupied orbitals (*i.e.*, they become better electron acceptors) (see Fig. 4).^10^ Important to note here is that molecular orbitals (MOs) involved in the H-bonds are delocalized over larger parts of the molecule and are not exclusively located on the frontier groups (Fig. 3d). So even if the charge at the frontier atoms does not change, a change in charge on other parts of the molecule causes shifts in the energies of the MOs [as long as the MO is asymmetric with respect to the molecule (see below)]. As such, we observe that upon changing from G1 to G2 and from C to C1, the larger accumulation of negative charge on the side of the H-bond acceptor group(s), that is, where the highest-occupied molecular orbitals (*σ*_HOMOs_) involved in the intermolecular H-bonds have their coefficients, destabilizes the *σ*_HOMOs_ (see Fig. 3d). At the same time, the larger accumulation of positive charge on the side of the H-bond donor group(s) stabilizes the lowest-unoccupied molecular orbitals (*σ*_LUMOs_) as these MOs have a coefficient on this side. Note that the *σ*_HOMO−1_ of the cytosine isosteres is not affected upon changing C to C1 because this MO is relatively symmetric and therefore there is no significant net effect of the change in molecular charge accumulation. Nevertheless, both the destabilization of the other *σ*_HOMOs_ and the stabilization of the *σ*_LUMOs_ reduces the energy gap (Δ*ε*) between the interacting orbitals and, therefore, results in better σ-orbital interactions in the case of enhanced charge accumulation (Fig. 4), that is, upon changing from G1 to G2 and from C to C1.^22^

## Conclusions

The binding strength of H-bonded aromatic dimers can be systematically tuned by varying the position of heteroatoms on the backside of the monomers while keeping the H-bond frontier atoms unchanged. These minimal non-frontier modifications can induce large interaction energy changes (ΔΔ*E*_int_ up to 6.5 kcal mol^−1^) although the charge of the H-bond frontier atoms is almost unaffected, as appears from our quantum-chemical analyses. Nevertheless, the grouping of the polar functional groups on one side of the monomer induces a larger accumulation of positive and negative charge in the molecule. This enhances the intermolecular electrostatic interactions, as well as the σ-orbital interactions by reducing the energy gaps between the interacting molecular orbitals. This is a manifestation of two effects: (i) electrostatic interactions are not only between frontier atoms but also between distant atoms (*i.e.*, long-range); (ii) molecular orbitals are delocalized over the molecule and their energies are therefore influenced by charge changes in non-frontier parts of the molecule. Our findings challenge the concept of explaining H-bond strengths by the frontier atoms, revealing that the strength of the electrostatic interactions, as well as the orbital interactions, are determined by the whole backbone of the H-bond donor and acceptor fragments.

## Conflicts of interest

There are no conflicts to declare.

## Supplementary Material

CP-026-D3CP05244C-s001

CP-026-D3CP05244C-s002
