# Supplementary material for: Multiple hydrogen-bonded dimers: are only the frontier atoms relevant?
Source: Phys Chem Chem Phys. 2023 Nov 30;26(15):11306–10. doi: 10.1039/d3cp05244c (PMC11022277; doi:10.1039/d3cp05244c)
Supplement: CP-026-D3CP05244C-s001 [file CP-026-D3CP05244C-s001.pdf]

Electronic Supplementary Material (ESI) for Physical Chemistry Chemical Physics.
This journal is © the Owner Societies 2023

Supporting Information

for

Multiple hydrogen-bonded dimers: Are only the frontier atoms relevant?

Celine Nieuwland,[a] David Almacellas,[b] Mac M. Veldhuizen,[a] Lucas de Azevedo Santos,[a] Jordi Poater,[b,c] and Célia Fonseca Guerra*[a]

[a] Department of Chemistry and Pharmaceutical Sciences, Amsterdam Institute for Molecular and Life Sciences (AIMMS), Vrije Universiteit Amsterdam, De Boelelaan 1108, 1081 HZ Amsterdam, The Netherlands. E-mail: c.fonsecaguerra@vu.nl | Homepage: https://www.theochem.nl/

[b] Departament de Química Inorgànica i Orgànica & Institut de Química Teòrica i Computacional, Universitat de Barcelona, Martí i Franquès 1-11, 08028 Barcelona, Catalonia, Spain.

[c] ICREA, Pg. Lluís Companys 23, 08010 Barcelona, Spain.

Table of Contents

Method S1. Computational details....2

Method S2. Hydrogen-bond and interaction energy analyses....3

Method S3. Voronoi deformation density (VDD) charges....5

Data S1. Equilibrium bond energy analysis of hydrogen-bonded **GC** base pair isosteres....6

Data S2. Complete EDA results as a function of the hydrogen-bond distance....6

Data S3. Atomic charges and relevant σ_{HOMO} and σ_{LUMO} orbitals of **G** and **C** isosteres....14

Data S4. Effect of **G** → **G1** isostere....16

Data S5. Cartesian coordinates and energies of optimized structures....17

Method S1. Computational details

All calculations were performed using the Amsterdam Density Functional (ADF) program as implemented in the Amsterdam Modeling Suite (AMS2022.101).[S1] The dispersion-corrected relativistic density functional theory (DFT-D) calculations were performed at the ZORA-BLYP-D3(BJ)[S2]/TZ2P level of theory, which has been proven to be adequate for studying hydrogen-bonded base pairs.[S3] The TZ2P basis set[S1c-e] consists of an uncontracted set of Slater-type orbitals (STOs) of triple-ζ quality for all atoms, augmented with two sets of polarization functions. The ZlmFit[S4a] density fitting scheme with the Becke[S4b] integration grid, at 'Excellent' quality, was used for geometry optimizations and subsequent (bonding) analyses. All optimized geometries have been checked to be energy minima by performing a vibrational frequency analysis (*i.e.,* no imaginary frequencies for minima, see Supporting Data S5).[S5]

The energy decomposition analyses (EDA, see Method S2 for a theoretical overview) as a function of the hydrogen-bond distance (see Fig. 2 in the main text and Data S2) were performed using the PyFrag 2019 program.[S6]

In Table S2, the performance of the following functionals have been tested in the computation of relative hydrogen-bond interaction energies: BLYP[2a–c]-D3(BJ)[2d–g], B3LYP[S7]-D3(BJ)[S2d–g], CAM-B3LYP[S8]-D3(BJ)[S2d–g], and ωB97X[S9]-D4. [S10]

[S1] (a) R. Rüger, M. Franchini, T. Trnka, A. Yakovlev, E. van Lenthe, P. Philipsen, T. van Vuren, B. Klumpers and T. Soini, *AMS2022.101*, SCM, Theoretical Chemistry, Vrije Universiteit Amsterdam, Amsterdam, 2022, www.scm.com; (b) C. Fonseca Guerra, J. G. Snijders, G. te Velde and E. J. Baerends, *Theor. Chem. Acc.*, 1998, **99**, 391; (c) G. te Velde, F. M. Bickelhaupt, E. J. Baerends, C. Fonseca Guerra, S. J. A. van Gisbergen, J. G. Snijders and T. Ziegler, *J. Comput. Chem.*, 2001, **22**, 931; (d) E. J. Baerends, D. E. Ellis and P. Ros, *Chem. Phys.,* 1973, **2**, 41; (e) E. van Lenthe and E. J. Baerends, *J. Comput. Chem.,* 2003, **24**, 1142.

[S2] (a) A. D. Becke, *Phys. Rev. A*, 1988, **38**, 3098; (b) C. Lee, W. Yang and R. G. Parr, *Phys. Rev. B,* 1988, **37**, 785; (c) Q. Wu and W. Yang, *J. Chem. Phys.*, 2002, **116**, 515; (d) S. Grimme, *J. Comput. Chem.,* 2004, **25**, 1463; (e) S. Grimme, *J. Comput. Chem.,* 2006, **27**, 1787; (f) S. Grimme, J. Antony, S. Ehrlich and H. Krieg, *J. Chem. Phys.,* 2010, **132**, 154104; (g) S. Grimme, S. Ehrlich and L. Goerigk, *J. Comput. Chem.,* 2011, **32**, 1456; (h) E. van Lenthe, A. Ehlers and E. J. Baerends, *J. Chem. Phys.* 1999, **110**, 8943.

[S3] (a) P. Vermeeren, L. P. Wolters, G. Paragi and C. Fonseca Guerra, *ChemPlusChem,* 2021, **86**, 812; (b) C. Fonseca Guerra, T. van der Wijst, J. Poater, M. Swart and F. M. Bickelhaupt, *Theor. Chem. Acc.,* 2010, **125**, 245.

[S4] (a) M. Franchini, P. H. T. Philipsen, E. van Lenthe and L. Visscher, *J. Chem. Theory Comput.,* 2014, **10**, 1994; (b) M. Franchini, P. H. T. Philipsen and L. Visscher, *J. Comput. Chem.,* 2013, **34**, 1819.

[S5] (a) S. K. Wolff, *Int. J. Quantum Chem.,* 2005, **104**, 645; (b) A. Bérces, R. M. Dickson, L. Fan, H. Jacobsen, D. P. Swerhone and T. Ziegler, *Comput. Phys. Commun.,* 1997, **100**, 247; (c) H. Jacobsen, A. Bérces, D. P. Swerhone and T. Ziegler, *Comput. Phys. Commun.,* 1997, **100**, 263.

[S6] (a) X. Sun, T. M. Soini, J. Poater, T. A. Hamlin and F. M. Bickelhaupt, *J. Comput. Chem.,* 2019, **40**, 2227; (b) X. Sun, T. Soini, L. P. Wolters, W.-J. van Zeist, C. Fonseca Guerra, T. A. Hamlin and F. M. Bickelhaupt, *PyFrag 2019*, Vrije Universiteit, Amsterdam, 2019.

[S7] P. J. Stephens, F. J. Devlin, C. F. Chabalowski and M. J. Frisch, *J. Phys. Chem.*, 1994, **98**, 11623.

[S8] T. Yanai, D. P. Tew and N. C. Handy, *Chem. Phys. Lett.,* 2004, **393**, 51.

[S9] J. D. Chai and M. Head-Gordon, *J. Chem. Phys.,* 2008, **128**, 084106.

[S10] (a) E. Caldeweyher, S. Ehlert, A. Hansen, H. Neugebauer, S. Spicher, C. Bannwarth, S. Grimme, *J. Chem. Phys.,* 2019, **150**, 154122; (b) E. Caldeweyher, J. M. Mewes, S. Ehlert, S. Grimme, *Phys. Chem. Chem. Phys.,* 2020, **22**, 8499.

Method S2. Hydrogen-bond and interaction energy analyses

In this work, the hydrogen-bond interactions of the various isosteres of the guanine–cytosine (**GC**) base pair were studied. The hydrogen-bond energy (ΔE) of the **GC** base pair (isostere) is formulated by Equation S1.

$$\Delta E = E(\mathbf{GC}) - E(\mathbf{G}) - E(\mathbf{C}) \qquad \text{(S1)}$$

In this equation, E(**GC**), E(**G**), and E(**C**) correspond to the electronic energies E of the hydrogen-bonded **GC** base pair isostere, and of the two separate nucleobases, each in their equilibrium geometry. Although all optimized **G** and **C** isosteres are not planar (*i.e.,* C_1 symmetric), the minimum energy structures of all **GC** base pair isosteres are C_s symmetric, that is, planar (see Supporting Data S5).

To understand the different components that determine the relative stabilities of the hydrogen-bonded base pairs, ΔE was partitioned according to the activation strain model (ASM)[S11] of reactivity and bonding into a strain and interaction energy component (Equation S2).

$$\Delta E = \Delta E_{\text{strain}} + \Delta E_{\text{int}} \qquad \text{(S2)}$$

In this decomposition, the strain energy (ΔE_{strain}) is the energy required to deform the nucleobase monomers from their equilibrium geometry to the geometry they acquire in the hydrogen-bonded pair. The interaction energy (ΔE_{int}) accounts for the stabilizing interaction between the two prepared (*i.e.,* deformed) bases.

ΔE_{int} can be further decomposed based on Kohn-Sham molecular orbital theory using a quantitative energy decomposition analysis (EDA).[S12] In the EDA, the total interaction energy (ΔE_{int}) is decomposed into components of electrostatic interaction (ΔV_{elstat}), Pauli repulsion (ΔE_{Pauli}), orbital interaction (ΔE_{oi}), and dispersion (ΔE_{disp}) (see Equation S3).

$$\Delta E_{\text{int}} = \Delta V_{\text{elstat}} + \Delta E_{\text{Pauli}} + \Delta E_{\text{oi}} + \Delta E_{\text{disp}} \qquad \text{(S3)}$$

[S11] (a) P. Vermeeren, S. C. C. van der Lubbe, C. Fonseca Guerra, F. M. Bickelhaupt and T. A. Hamlin, *Nat. Protoc.,* 2020, **15**, 649; (b) F. M. Bickelhaupt and K. N. Houk, *Angew. Chem. Int. Ed.,* 2017, **56**, 10070; *Angew. Chem.,* 2017, **129**, 10204; (d) P. Vermeeren, T. A. Hamlin and F. M. Bickelhaupt, *Chem. Commun.,* 2021, **57**, 5880.

[S12] (a) F. M. Bickelhaupt and E. J. Baerends, in *Reviews in Computational Chemistry*, ed. K. B. Lipkowitz and D. B. Boyd, Wiley-VCH, New York, 2000, **15**, pp. 1–86; (b) T. A. Hamlin, P. Vermeeren, C. Fonseca Guerra, F. M. Bickelhaupt, in *Complementary Bonding Analysis*, ed. S. Grabowsky, De Gruyter, Berlin, 2021, **8**, pp. 199–212.

Here, ΔV_{elstat} comprises the (usually attractive) classical electrostatic interactions between the unperturbed charge distributions of the prepared (*i.e.,* deformed) interacting monomers. ΔE_{Pauli} accounts for the destabilizing interactions resulting from overlapping closed-shell orbitals and accounts for any steric repulsion. The ΔE_{oi} term comprises i) charge transfer between the interacting monomers (*i.e.,* donor–acceptor interactions between occupied and unoccupied orbitals on the interacting monomer, including HOMO–LUMO interactions) which occurs for hydrogen-bonded dimers in the σ-electronic system, and ii) mutual polarization of the monomers (*i.e.,* empty–occupied orbital mixing on one monomer due to the presence of the other monomer) which occurs for hydrogen-bonded dimers in the π-electronic system. Due to the planar (C_s) symmetry of the hydrogen-bonded pairs, the total orbital interaction term (ΔE_{oi}) can be decomposed into these contributions stemming from the σ-charge transfer (ΔE_{oi}^{σ}) and π-polarization (ΔE_{oi}^{π}) orbital interactions (Equation S4). Lastly, the ΔE_{disp} term includes a dispersion energy correction because of the use of Grimme's D3 dispersion correction in the computations (see Method S1 for the full computational details).

$$\Delta E_{oi} = \Delta E_{oi}^{\sigma} + \Delta E_{oi}^{\pi} \qquad \text{(S4)}$$

Besides in the equilibrium geometries of the **GC** base pair (isosteres) (see Data S1), the interaction energies ΔE_{int} were also analyzed as a function of the middle hydrogen-bond distance $r_{N(H)\cdots N}$ of the **GC** base pairs (see Fig. 2 in the main text and Data S2). In this approach, the hydrogen-bond distances were varied over a certain hydrogen-bond distance interval while keeping the monomers *frozen* in the geometry that they acquire in the optimized **GC** base pair (isostere). The advantage of this approach is that we can compare the base pairs at similar hydrogen-bond distances while preserving the other geometrical characteristics. This allows us to differentiate between interaction terms that are intrinsically more stabilizing, from the interaction terms that are simply enhanced by the shortened hydrogen-bond distances. In other words, comparing the base pairs at similar hydrogen-bond distances allows us to identify which interaction energy term (ΔV_{elstat}, ΔE_{Pauli}, ΔE_{oi}, or a combination thereof) causes the relative hydrogen-bond strengths. This approach, in which the monomer geometries approach each other as frozen fragments, has been demonstrated before to yield identical results compared to the approach in which the geometries of the hydrogen-bonded monomers are allowed to relax (*i.e.,* optimize) at each step of a consistent hydrogen-bond distance.[S13]

[S13] S. C. C. van der Lubbe, F. Zaccaria, X. Sun and C. Fonseca Guerra, *J. Am. Chem. Soc.,* 2019, **141**, 4878.

Method S3. Voronoi deformation density (VDD) charges

The Voronoi Deformation Density (VDD) charge analysis allows for the quantification of the flow of electronic charge as a direct consequence of chemical-bond formation.[S14] VDD atomic charges (Q) are computed by the spatial integration of the deformation density over the Voronoi cell of atom A, which is the space defined by the bond midplanes on and perpendicular to all bond axes between this atom A and its neighboring atoms (see Equation S5).

$$Q = -\int_{\text{Voronoi cell of A}} [\rho(\mathbf{r}) - \textstyle\sum_i \rho_i(\mathbf{r})]\,d\mathbf{r} \qquad \text{(S5)}$$

Herein, the deformation density $\Delta\rho(\mathbf{r}) = [\rho(\mathbf{r}) - \sum_i \rho_i(\mathbf{r})]$ is the density change going from a superposition of the original atomic densities at the positions of the molecule to the actual density of that molecule. This atomic or so-called *promolecular* density is defined as the sum of the (spherically averaged) ground-state atomic densities $\sum_i \rho_i(\mathbf{r})$. This is the fictitious state in which the charge density has not been affected by chemical bonding and in which all atoms have zero charge. Q in Equation S5 then represents the amount of charge that, due to chemical bonding, flows to a position closer to nucleus A ($Q < 0$) or to a position further away from nucleus A ($Q > 0$).

[S14] (a) C. Nieuwland, P. Vermeeren, F. M. Bickelhaupt and C. Fonseca Guerra, *J. Comput. Chem.,* 2023, **44**, 2108; (b) C. Fonseca Guerra, J. W. Handgraaf, E. J. Baerends and F. M. Bickelhaupt, *J. Comput. Chem.,* 2004, **25**, 189; (c) O. A. Stasyuk, H. Szatylowicz, T. M. Krygowski and C. Fonseca Guerra, *Phys. Chem. Chem. Phys.,* 2016, **18**, 11624.

Data S1. Equilibrium bond energy analysis of hydrogen-bonded **GC** base pair isosteres

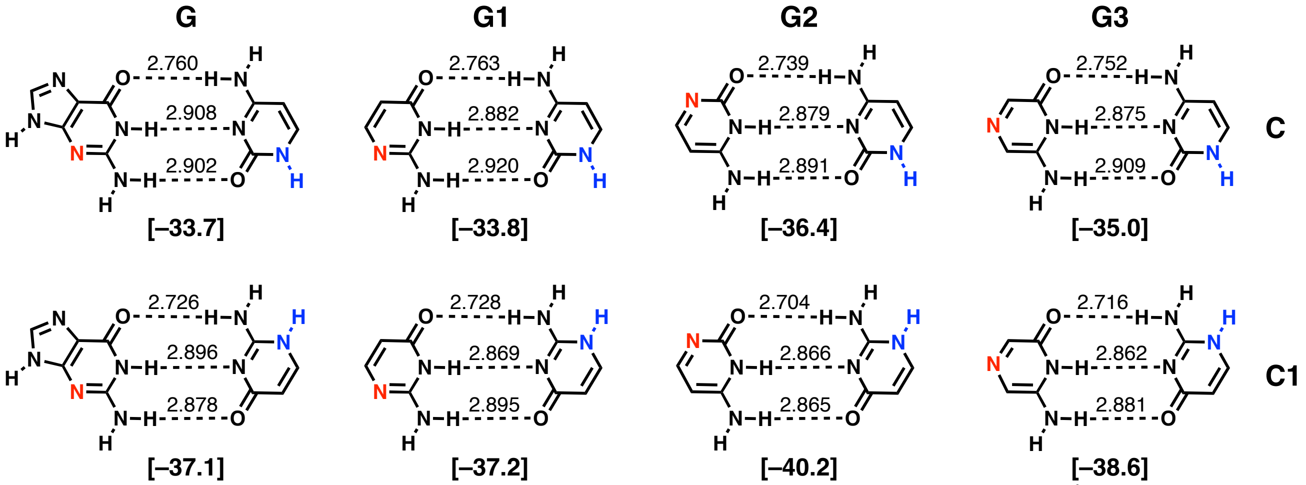

Figure S1. GC base-pair (isosteres) with equilibrium hydrogen-bond distances (in Å) and interaction energies ΔE_{int} (shown in between brackets in kcal mol^{-1}) computed at ZORA-BLYP-D3(BJ)/TZ2P.

Table S1. Decomposition of the equilibrium hydrogen-bond energy ΔE (in kcal mol^{-1}) of the planar **GC** hydrogen-bonded base pair (isosteres) of Figure S1.[a]

	ΔE	$\Delta E_{\mathrm{strain}}$	ΔE_{int}	$\Delta V_{\mathrm{elstat}}$	$\Delta E_{\mathrm{Pauli}}$	ΔE_{oi}[b]	$\Delta E_{\mathrm{oi}}^{\sigma}$	$\Delta E_{\mathrm{oi}}^{\pi}$	ΔE_{disp}
**GC**	–29.7	3.9	–33.7	–46.5	49.2	–30.2	–25.9	–4.2	–6.2
**G1C**	–30.0	3.8	–33.8	–47.3	50.6	–30.9	–26.6	–4.2	–6.2
**G2C**	–32.1	4.2	–36.4	–50.3	53.6	–33.4	–28.6	–4.8	–6.3
**G3C**[c]	–30.7	4.3	–35.0	–49.2	53.1	–32.6	–27.9	–4.7	–6.3
**GC1**	–31.7	5.3	–37.1	–50.6	53.6	–33.8	–28.9	–4.8	–6.3
**G1C1**	–31.9	5.3	–37.2	–51.4	55.1	–34.5	–29.7	–4.8	–6.3
**G2C1**	–34.3	5.9	–40.2	–54.9	58.6	–37.5	–32.0	–5.5	–6.4
**G3C1**[c]	–32.7	5.9	–38.6	–53.5	58.0	–36.6	–31.2	–5.4	–6.4

[a] Computed at ZORA-BLYP-D3(BJ)/TZ2P. [b] The orbital interaction can be decomposed into components of the σ- and π-orbital interactions due to the C_s symmetry of the base pairs: $\Delta E_{\mathrm{oi}} = \Delta E_{\mathrm{oi}}^{\sigma} + \Delta E_{\mathrm{oi}}^{\pi}$. [b] The third possible guanine isostere (**G3**) yields an average effect on the hydrogen-bond strength compared to **G1** and **G2** because it has a molecular charge accumulation which is in between that of **G1** and **G2** (molecular dipole moments: 8.8 (**G2**), 6.9 (**G3**), 5.0 (**G1**) Debye).

Table S2. Agreement of different density functionals in the decomposition of the interaction energy ΔE_{int} (in kcal mol^{-1}) of the equilibrium hydrogen-bonded base pair isosteres **G1C** and **G2C1**.[a,b]

	**G1C**					**G2C1**					
	ΔE_{int}	$\Delta V_{\mathrm{elstat}}$	$\Delta E_{\mathrm{Pauli}}$	ΔE_{oi}	ΔE_{disp}	ΔE_{int}	$\Delta V_{\mathrm{elstat}}$	$\Delta E_{\mathrm{Pauli}}$	ΔE_{oi}	ΔE_{disp}	$\Delta\Delta E_{\mathrm{int}}$
**BLYP-D3(BJ)**[c]	–33.8	–47.3	50.6	–30.9	–6.2	–40.2	–54.9	58.6	–37.5	–6.4	–6.4
**B3LYP-D3(BJ)**	–35.0	–47.6	46.7	–28.9	–5.2	–41.5	–55.4	54.4	–35.2	–5.3	–6.5
**CAM-B3LYP-D3(BJ)**	–35.8	–48.5	43.9	–27.9	–3.2	–42.4	–56.5	51.5	–34.2	–3.3	–6.6
**ωB97X-D4**	–35.1	–47.8	40.8	–27.2	–1.0	–41.6	–55.6	48.3	–33.3	–1.0	–6.5

[a] Computed at ZORA-{Functional}/TZ2P//ZORA-BLYP-D3(BJ)/TZ2P. [b] $\Delta\Delta E_{\mathrm{int}} = \Delta E_{\mathrm{int}}$(**G2C1**) – ΔE_{int}(**G1C**). [c] Level of theory used in this work.

Data S2. Complete EDA results as a function of the hydrogen-bond distance

Table S3. The following tables contain the complete energy decomposition analysis (EDA) results in kcal mol^{-1} (see Method S2 for the computational details) of the hydrogen-bond interaction of the **GC** base pair isosteres as a function of the middle H-bond distance $r_{N(H)\cdots N}$ (step size of 0.01 Å) in which the monomers approach each other as frozen blocks. All computed at ZORA-BLYP-D3(BJ)/TZ2P in C_s symmetry.

**Total interaction energy ΔE_{int}**						
$r_{N(H)\cdots N}$ **(Å)**	**GC**	**G1C**	**G2C**	**GC1**	**G1C1**	**G2C1**
2.80	–32.8	–33.3	–35.9	–36.4	–36.8	–39.8
2.81	–33.0	–33.4	–36.0	–36.5	–36.9	–39.9
2.82	–33.1	–33.5	–36.1	–36.7	–37.0	–40.0
2.83	–33.2	–33.6	–36.2	–36.8	–37.1	–40.1
2.84	–33.3	–33.7	–36.3	–36.9	–37.1	–40.1
2.85	–33.4	–33.7	–36.3	–36.9	–37.2	–40.2
2.86	–33.5	–33.8	–36.4	–37.0	–37.2	–40.2
2.87	–33.6	–33.8	–36.4	–37.0	–37.2	–40.2
2.88	–33.6	–33.8	–36.4	–37.1	–37.2	–40.2
2.89	–33.7	–33.8	–36.4	–37.1	–37.2	–40.2
2.90	–33.7	–33.8	–36.4	–37.1	–37.1	–40.1
2.91	–33.7	–33.8	–36.3	–37.1	–37.1	–40.1
2.92	–33.7	–33.7	–36.3	–37.1	–37.0	–40.0
2.93	–33.6	–33.7	–36.2	–37.0	–37.0	–39.9
2.94	–33.6	–33.6	–36.2	–37.0	–36.9	–39.8
2.95	–33.6	–33.5	–36.1	–36.9	–36.8	–39.7
2.96	–33.5	–33.5	–36.0	–36.8	–36.7	–39.6
2.97	–33.5	–33.4	–35.9	–36.8	–36.6	–39.5
2.98	–33.4	–33.3	–35.8	–36.7	–36.5	–39.4
2.99	–33.3	–33.2	–35.7	–36.6	–36.4	–39.3
3.00	–33.2	–33.1	–35.6	–36.5	–36.2	–39.1
3.01	–33.1	–32.9	–35.4	–36.3	–36.1	–39.0
3.02	–33.0	–32.8	–35.3	–36.2	–35.9	–38.8
3.03	–32.9	–32.7	–35.1	–36.1	–35.8	–38.6
3.04	–32.8	–32.5	–35.0	–35.9	–35.6	–38.5
3.05	–32.6	–32.4	–34.8	–35.8	–35.5	–38.3
3.06	–32.5	–32.3	–34.7	–35.6	–35.3	–38.1
3.07	–32.4	–32.1	–34.5	–35.5	–35.1	–37.9
3.08	–32.2	–31.9	–34.4	–35.3	–35.0	–37.7
3.09	–32.1	–31.8	–34.2	–35.2	–34.8	–37.5
3.10	–31.9	–31.6	–34.0	–35.0	–34.6	–37.3

Electrostatic interaction ΔV_{elstat}						
$r_{N(H)\cdots N}$ (Å)	**GC**	**G1C**	**G2C**	**GC1**	**G1C1**	**G2C1**
2.80	–56.3	–54.6	–57.7	–59.8	–58.0	–61.5
2.81	–55.3	–53.7	–56.7	–58.7	–57.0	–60.5
2.82	–54.3	–52.7	–55.7	–57.7	–56.0	–59.4
2.83	–53.3	–51.8	–54.7	–56.7	–55.0	–58.4
2.84	–52.4	–50.9	–53.8	–55.7	–54.1	–57.4
2.85	–51.5	–50.0	–52.8	–54.7	–53.1	–56.4
2.86	–50.6	–49.1	–51.9	–53.8	–52.2	–55.5
2.87	–49.7	–48.3	–51.0	–52.9	–51.3	–54.6
2.88	–48.8	–47.4	–50.2	–52.0	–50.5	–53.6
2.89	–48.0	–46.6	–49.3	–51.1	–49.6	–52.8
2.90	–47.2	–45.8	–48.5	–50.2	–48.8	–51.9
2.91	–46.3	–45.1	–47.7	–49.4	–48.0	–51.0
2.92	–45.6	–44.3	–46.9	–48.6	–47.2	–50.2
2.93	–44.8	–43.6	–46.1	–47.8	–46.4	–49.4
2.94	–44.1	–42.8	–45.4	–47.0	–45.7	–48.6
2.95	–43.3	–42.1	–44.6	–46.2	–45.0	–47.8
2.96	–42.6	–41.5	–43.9	–45.5	–44.2	–47.1
2.97	–41.9	–40.8	–43.2	–44.8	–43.5	–46.3
2.98	–41.2	–40.1	–42.5	–44.0	–42.8	–45.6
2.99	–40.6	–39.5	–41.8	–43.3	–42.2	–44.9
3.00	–39.9	–38.9	–41.2	–42.7	–41.5	–44.2
3.01	–39.3	–38.2	–40.5	–42.0	–40.9	–43.6
3.02	–38.6	–37.6	–39.9	–41.4	–40.2	–42.9
3.03	–38.0	–37.1	–39.3	–40.7	–39.6	–42.2
3.04	–37.4	–36.5	–38.7	–40.1	–39.0	–41.6
3.05	–36.9	–35.9	–38.1	–39.5	–38.4	–41.0
3.06	–36.3	–35.4	–37.5	–38.9	–37.9	–40.4
3.07	–35.7	–34.8	–37.0	–38.3	–37.3	–39.8
3.08	–35.2	–34.3	–36.4	–37.7	–36.7	–39.2
3.09	–34.7	–33.8	–35.9	–37.2	–36.2	–38.7
3.10	–34.1	–33.3	–35.4	–36.6	–35.7	–38.1

Pauli repulsion ΔE_{Pauli}						
$r_{N(H)\cdots N}$ (Å)	GC	G1C	G2C	GC1	G1C1	G2C1
2.80	70.1	65.9	69.2	73.2	68.9	72.7
2.81	67.8	63.8	67.0	70.9	66.7	70.4
2.82	65.6	61.8	64.9	68.6	64.6	68.1
2.83	63.5	59.8	62.8	66.4	62.5	66.0
2.84	61.5	57.9	60.8	64.2	60.5	63.8
2.85	59.5	56.0	58.8	62.2	58.6	61.8
2.86	57.6	54.3	56.9	60.2	56.7	59.8
2.87	55.7	52.5	55.1	58.3	54.9	57.9
2.88	54.0	50.9	53.4	56.4	53.2	56.1
2.89	52.2	49.2	51.7	54.6	51.5	54.3
2.90	50.6	47.7	50.0	52.8	49.8	52.5
2.91	48.9	46.1	48.4	51.1	48.2	50.9
2.92	47.4	44.7	46.9	49.5	46.7	49.2
2.93	45.9	43.3	45.4	47.9	45.2	47.7
2.94	44.4	41.9	43.9	46.4	43.8	46.1
2.95	43.0	40.6	42.5	44.9	42.4	44.7
2.96	41.6	39.3	41.2	43.5	41.1	43.2
2.97	40.3	38.0	39.9	42.1	39.8	41.9
2.98	39.0	36.8	38.6	40.7	38.5	40.5
2.99	37.8	35.7	37.4	39.4	37.3	39.3
3.00	36.6	34.5	36.2	38.2	36.1	38.0
3.01	35.4	33.4	35.0	37.0	35.0	36.8
3.02	34.3	32.4	33.9	35.8	33.9	35.6
3.03	33.2	31.4	32.9	34.7	32.8	34.5
3.04	32.1	30.4	31.8	33.6	31.8	33.4
3.05	31.1	29.4	30.8	32.5	30.8	32.4
3.06	30.1	28.5	29.8	31.5	29.8	31.3
3.07	29.2	27.6	28.9	30.5	28.9	30.4
3.08	28.2	26.7	28.0	29.5	28.0	29.4
3.09	27.4	25.9	27.1	28.6	27.1	28.5
3.10	26.5	25.1	26.3	27.7	26.2	27.6

Total orbital interaction ΔE_{oi}						
$r_{N(H)\cdots N}$ (Å)	GC	G1C	G2C	GC1	G1C1	G2C1
2.80	–39.8	–38.0	–40.7	–43.0	–41.1	–44.3
2.81	–38.8	–37.0	–39.7	–41.9	–40.1	–43.2
2.82	–37.8	–36.1	–38.7	–40.8	–39.1	–42.1
2.83	–36.8	–35.2	–37.7	–39.8	–38.1	–41.1
2.84	–35.9	–34.3	–36.8	–38.8	–37.1	–40.0
2.85	–35.0	–33.4	–35.9	–37.8	–36.2	–39.1
2.86	–34.1	–32.6	–35.0	–36.9	–35.3	–38.1
2.87	–33.2	–31.8	–34.1	–36.0	–34.5	–37.2
2.88	–32.4	–31.0	–33.3	–35.1	–33.6	–36.3
2.89	–31.6	–30.2	–32.5	–34.2	–32.8	–35.4
2.90	–30.8	–29.5	–31.7	–33.4	–32.0	–34.5
2.91	–30.1	–28.8	–30.9	–32.6	–31.2	–33.7
2.92	–29.3	–28.1	–30.2	–31.8	–30.5	–32.9
2.93	–28.6	–27.4	–29.4	–31.0	–29.7	–32.1
2.94	–27.9	–26.7	–28.7	–30.3	–29.0	–31.3
2.95	–27.2	–26.1	–28.0	–29.5	–28.3	–30.6
2.96	–26.6	–25.5	–27.4	–28.8	–27.7	–29.9
2.97	–25.9	–24.8	–26.7	–28.1	–27.0	–29.2
2.98	–25.3	–24.3	–26.1	–27.5	–26.4	–28.5
2.99	–24.7	–23.7	–25.5	–26.8	–25.7	–27.8
3.00	–24.1	–23.1	–24.9	–26.2	–25.1	–27.2
3.01	–23.5	–22.6	–24.3	–25.6	–24.6	–26.5
3.02	–23.0	–22.0	–23.7	–25.0	–24.0	–25.9
3.03	–22.4	–21.5	–23.2	–24.4	–23.4	–25.3
3.04	–21.9	–21.0	–22.6	–23.8	–22.9	–24.7
3.05	–21.4	–20.5	–22.1	–23.3	–22.4	–24.2
3.06	–20.9	–20.1	–21.6	–22.7	–21.9	–23.6
3.07	–20.4	–19.6	–21.1	–22.2	–21.4	–23.1
3.08	–19.9	–19.1	–20.6	–21.7	–20.9	–22.6
3.09	–19.5	–18.7	–20.1	–21.2	–20.4	–22.1
3.10	–19.0	–18.3	–19.7	–20.7	–19.9	–21.6

	σ-orbital interaction ΔE_{oi}^{σ}					
$r_{N(H)\cdots N}$ (Å)	GC	G1C	G2C	GC1	G1C1	G2C1
2.80	–34.4	–32.9	–35.0	–37.1	–35.5	–38.0
2.81	–33.5	–32.1	–34.1	–36.1	–34.6	–37.0
2.82	–32.7	–31.2	–33.2	–35.2	–33.7	–36.0
2.83	–31.8	–30.4	–32.4	–34.3	–32.8	–35.1
2.84	–31.0	–29.6	–31.6	–33.4	–32.0	–34.2
2.85	–30.2	–28.9	–30.8	–32.5	–31.2	–33.4
2.86	–29.4	–28.2	–30.0	–31.7	–30.4	–32.5
2.87	–28.6	–27.4	–29.2	–30.9	–29.6	–31.7
2.88	–27.9	–26.7	–28.5	–30.1	–28.9	–30.9
2.89	–27.2	–26.1	–27.8	–29.4	–28.2	–30.1
2.90	–26.5	–25.4	–27.1	–28.6	–27.5	–29.4
2.91	–25.8	–24.8	–26.4	–27.9	–26.8	–28.7
2.92	–25.2	–24.2	–25.7	–27.2	–26.1	–27.9
2.93	–24.6	–23.6	–25.1	–26.5	–25.5	–27.3
2.94	–23.9	–23.0	–24.5	–25.9	–24.8	–26.6
2.95	–23.3	–22.4	–23.9	–25.2	–24.2	–25.9
2.96	–22.8	–21.8	–23.3	–24.6	–23.6	–25.3
2.97	–22.2	–21.3	–22.7	–24.0	–23.1	–24.7
2.98	–21.6	–20.8	–22.1	–23.4	–22.5	–24.1
2.99	–21.1	–20.3	–21.6	–22.8	–22.0	–23.5
3.00	–20.6	–19.8	–21.1	–22.3	–21.4	–22.9
3.01	–20.1	–19.3	–20.6	–21.7	–20.9	–22.4
3.02	–19.6	–18.8	–20.1	–21.2	–20.4	–21.9
3.03	–19.1	–18.4	–19.6	–20.7	–19.9	–21.3
3.04	–18.6	–17.9	–19.1	–20.2	–19.4	–20.8
3.05	–18.2	–17.5	–18.7	–19.7	–19.0	–20.3
3.06	–17.8	–17.1	–18.2	–19.2	–18.5	–19.9
3.07	–17.3	–16.7	–17.8	–18.8	–18.1	–19.4
3.08	–16.9	–16.3	–17.4	–18.3	–17.7	–18.9
3.09	–16.5	–15.9	–17.0	–17.9	–17.3	–18.5
3.10	–16.1	–15.5	–16.6	–17.5	–16.9	–18.1

	π-orbital interaction ΔE_{oi}^{π}					
$r_{N(H)\cdots N}$ (Å)	GC	G1C	G2C	GC1	G1C1	G2C1
2.80	–5.4	–5.1	–5.7	–5.9	–5.6	–6.3
2.81	–5.2	–4.9	–5.6	–5.8	–5.5	–6.2
2.82	–5.1	–4.8	–5.5	–5.7	–5.4	–6.1
2.83	–5.0	–4.7	–5.4	–5.5	–5.3	–6.0
2.84	–4.9	–4.6	–5.3	–5.4	–5.1	–5.8
2.85	–4.8	–4.5	–5.1	–5.3	–5.0	–5.7
2.86	–4.7	–4.4	–5.0	–5.2	–4.9	–5.6
2.87	–4.6	–4.3	–4.9	–5.1	–4.8	–5.5
2.88	–4.5	–4.3	–4.8	–5.0	–4.7	–5.4
2.89	–4.4	–4.2	–4.7	–4.9	–4.6	–5.3
2.90	–4.3	–4.1	–4.6	–4.8	–4.5	–5.2
2.91	–4.2	–4.0	–4.5	–4.7	–4.5	–5.0
2.92	–4.1	–3.9	–4.4	–4.6	–4.4	–4.9
2.93	–4.1	–3.8	–4.4	–4.5	–4.3	–4.9
2.94	–4.0	–3.8	–4.3	–4.4	–4.2	–4.8
2.95	–3.9	–3.7	–4.2	–4.3	–4.1	–4.7
2.96	–3.8	–3.6	–4.1	–4.2	–4.0	–4.6
2.97	–3.7	–3.5	–4.0	–4.2	–4.0	–4.5
2.98	–3.7	–3.5	–3.9	–4.1	–3.9	–4.4
2.99	–3.6	–3.4	–3.9	–4.0	–3.8	–4.3
3.00	–3.5	–3.3	–3.8	–3.9	–3.7	–4.2
3.01	–3.5	–3.3	–3.7	–3.8	–3.7	–4.2
3.02	–3.4	–3.2	–3.7	–3.8	–3.6	–4.1
3.03	–3.3	–3.1	–3.6	–3.7	–3.5	–4.0
3.04	–3.3	–3.1	–3.5	–3.6	–3.5	–3.9
3.05	–3.2	–3.0	–3.5	–3.6	–3.4	–3.9
3.06	–3.1	–3.0	–3.4	–3.5	–3.3	–3.8
3.07	–3.1	–2.9	–3.3	–3.4	–3.3	–3.7
3.08	–3.0	–2.9	–3.3	–3.4	–3.2	–3.6
3.09	–3.0	–2.8	–3.2	–3.3	–3.1	–3.6
3.10	–2.9	–2.8	–3.1	–3.3	–3.1	–3.5

Dispersion energy ΔE_{disp}						
$r_{N(H)\cdots N}$ (Å)	GC	G1C	G2C	GC1	G1C1	G2C1
2.80	–6.8	–6.6	–6.7	–6.8	–6.7	–6.8
2.81	–6.7	–6.6	–6.7	–6.8	–6.6	–6.7
2.82	–6.7	–6.5	–6.6	–6.7	–6.6	–6.6
2.83	–6.6	–6.5	–6.5	–6.7	–6.5	–6.6
2.84	–6.6	–6.4	–6.5	–6.6	–6.5	–6.5
2.85	–6.5	–6.4	–6.4	–6.6	–6.4	–6.5
2.86	–6.5	–6.3	–6.4	–6.5	–6.4	–6.4
2.87	–6.4	–6.3	–6.3	–6.4	–6.3	–6.4
2.88	–6.4	–6.2	–6.3	–6.4	–6.3	–6.3
2.89	–6.3	–6.2	–6.2	–6.3	–6.2	–6.3
2.90	–6.3	–6.1	–6.2	–6.3	–6.2	–6.2
2.91	–6.2	–6.1	–6.1	–6.2	–6.1	–6.2
2.92	–6.1	–6.0	–6.1	–6.2	–6.1	–6.1
2.93	–6.1	–6.0	–6.0	–6.1	–6.0	–6.1
2.94	–6.0	–5.9	–6.0	–6.1	–6.0	–6.0
2.95	–6.0	–5.9	–5.9	–6.0	–5.9	–6.0
2.96	–5.9	–5.8	–5.9	–6.0	–5.9	–5.9
2.97	–5.9	–5.8	–5.8	–5.9	–5.8	–5.9
2.98	–5.8	–5.7	–5.8	–5.9	–5.8	–5.8
2.99	–5.8	–5.7	–5.7	–5.8	–5.7	–5.8
3.00	–5.7	–5.6	–5.7	–5.8	–5.7	–5.7
3.01	–5.7	–5.6	–5.6	–5.7	–5.6	–5.7
3.02	–5.6	–5.5	–5.6	–5.7	–5.6	–5.6
3.03	–5.6	–5.5	–5.5	–5.6	–5.5	–5.6
3.04	–5.5	–5.4	–5.5	–5.6	–5.5	–5.5
3.05	–5.5	–5.4	–5.4	–5.5	–5.4	–5.5
3.06	–5.5	–5.3	–5.4	–5.5	–5.4	–5.4
3.07	–5.4	–5.3	–5.3	–5.4	–5.3	–5.4
3.08	–5.4	–5.2	–5.3	–5.4	–5.3	–5.3
3.09	–5.3	–5.2	–5.3	–5.3	–5.2	–5.3
3.10	–5.3	–5.1	–5.2	–5.3	–5.2	–5.2

Data S3. Atomic charges and relevant σHOMO and σLUMO orbitals of **G** and **C** isosteres

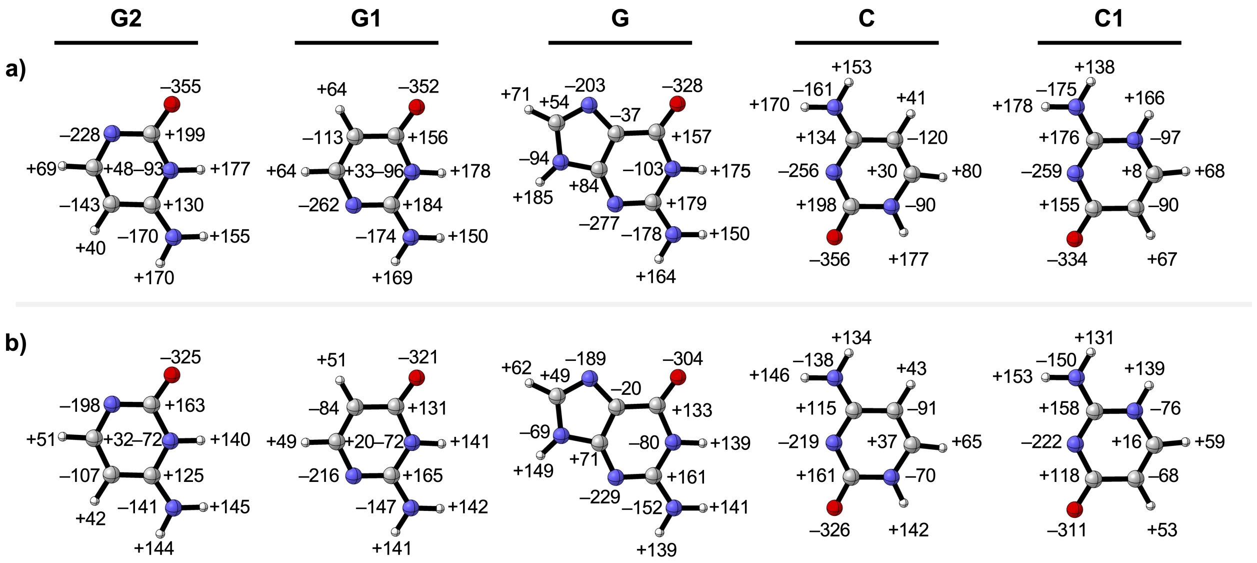

Figure S2. a) Voronoi deformation density (VDD) and b) Hirshfeld atomic charges Q (in milli-electrons) of the isolated guanine (**G**, **G1**, and **G2**) and cytosine (**C** and **C1**) isosteres in the geometry within the dimer with **C** or **G1**, respectively. Computed at ZORA-BLYP-D3(BJ)/TZ2P. Both charge-analysis methods demonstrate that the charge on the frontier atoms does not significantly change among the various **G** or **C** isosteres.

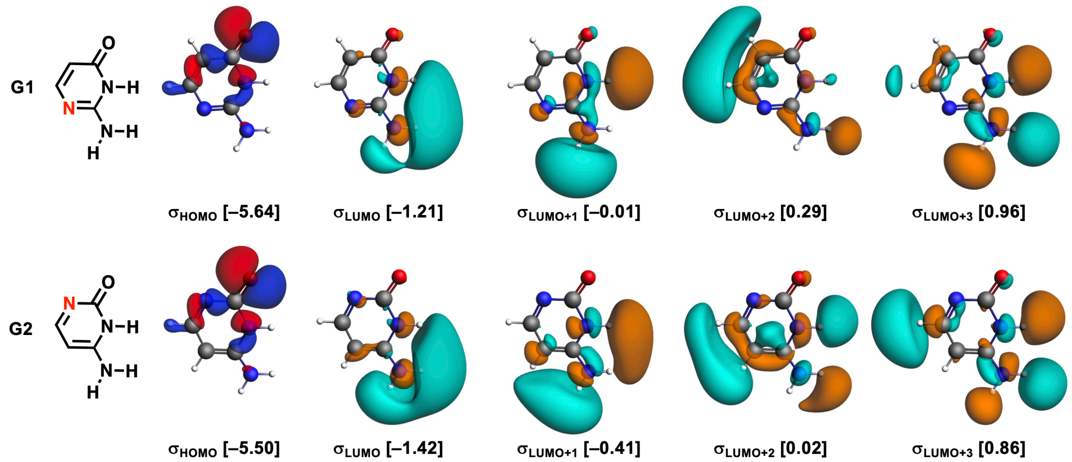

Figure S3. Isosurfaces (at 0.03 au) and corresponding energies ε (in eV) of the empty (σ_{LUMO}) and filled (σ_{HOMO}) orbitals involved in the hydrogen bonding of the **G1** and **G2** bases in the base pair with **C**. Computed at ZORA-BLYP-D3(BJ)/TZ2P.

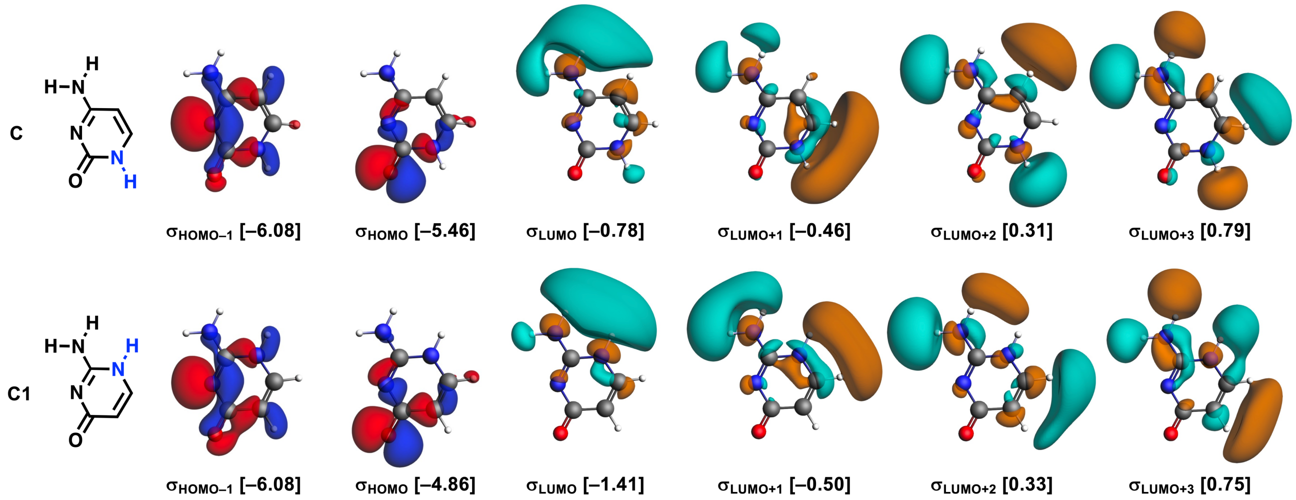

Figure S4. Isosurfaces (at 0.03 au) and corresponding energies ε (in eV) of the empty (σ_{LUMO}) and filled (σ_{HOMO}) orbitals involved in the H-bonding of the **C** and **C1** bases in the base pair with **G1**. Computed at ZORA-BLYP-D3(BJ)/TZ2P.

Table S4. Orbital overlaps *S* (in au) between the empty (σ_{LUMO}) and filled (σ_{HOMO}) orbitals involved in the H-bonding of the cytosine (**C** and **C1**)–guanine (**G1** and **G2**) base-pair isostere equilibrium geometries.[a]

		**G1**			**G2**		
		σ_{LUMO+1}	σ_{LUMO}	σ_{HOMO}	σ_{LUMO+1}	σ_{LUMO}	σ_{HOMO}
	σ_{LUMO}			0.07			0.06
**C**	σ_{HOMO}	0.03	0.14		0.08	0.13	
	σ_{HOMO-1}	0.24	0.22		0.21	0.18	
	σ_{LUMO}			0.06			0.05
**C1**	σ_{HOMO}	0.06	0.17		0.10	0.15	
	σ_{HOMO-1}	0.23	0.21		0.20	0.17	

[a] Computed at ZORA-BLYP-D3(BJ)/TZ2P. See Fig. 3d in the main text for the corresponding isosurfaces.

Data S4. Effect of **G** → **G1** isostere

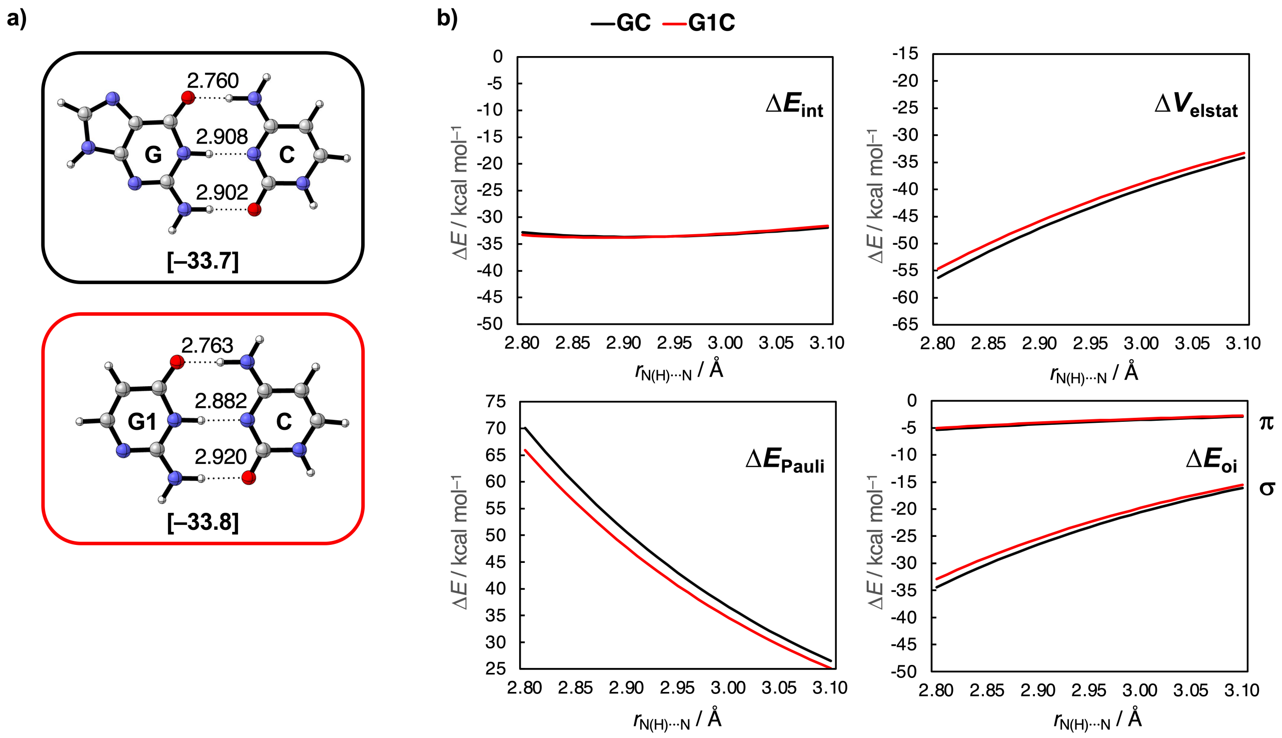

Figure S5. a) Equilibrium hydrogen-bonded base pairs **GC** and **G1C** with hydrogen-bond distances (in Å) and interaction energy ΔE_{int} (in kcal mol^{-1} in between brackets) and b) decomposed interaction energy terms as a function of the middle hydrogen bond distance $r_{N(H)\cdots N}$ (in Å, step size of 0.01 Å). All computed at ZORA-BLYP-D3(BJ)/TZ2P.

Data S5. Cartesian coordinates and energies of optimized structures

Below is a list consisting of the Cartesian coordinates (in Å), total bond energies *E*, enthalpies *H*, Gibbs free energies *G* (in kcal mol^{-1}), and the number of imaginary frequencies (N_{imag}) of all reported structures optimized at the ZORA-BLYP-D3(BJ)/TZ2P level. The ball-and-stick structures of the optimized structures in this work were made with the CYLview20[S15] visualization software.

GC

E: −4396.42
H: −4254.65
G: −4291.92
N_{imag} = 0

N	0.59867372	-2.59561488	0.00000000
O	1.05345131	2.01711820	0.00000000
N	0.92872482	-0.28881279	0.00000000
C	1.47019236	-1.55836777	0.00000000
N	2.78382843	-1.78973225	0.00000000
C	3.51388235	-0.65337132	0.00000000
C	3.07174580	0.68421038	0.00000000
C	1.66043411	0.92575481	0.00000000
N	4.89206367	-0.56794455	0.00000000
C	5.21678217	0.79120432	0.00000000
N	4.15308827	1.56345305	0.00000000
H	-0.42092927	-2.46773860	0.00000000
H	0.99421096	-3.52509469	0.00000000
H	-0.10742681	-0.18798821	0.00000000
H	5.52758939	-1.35729897	0.00000000
H	6.24625629	1.12769418	0.00000000
O	-2.28007418	-2.22739882	0.00000000
N	-1.68796952	2.34019800	0.00000000
N	-1.95907410	0.05644013	0.00000000
C	-3.94752695	1.46543337	0.00000000
C	-2.72640556	-1.06813212	0.00000000
N	-4.13680265	-0.88849832	0.00000000
C	-4.71981554	0.34599028	0.00000000
C	-2.51392107	1.28373228	0.00000000
H	-4.39339623	2.45391355	0.00000000
H	-4.69181664	-1.73813395	0.00000000
H	-5.80548499	0.37278708	0.00000000
H	-0.64857318	2.21087453	0.00000000
H	-2.07170700	3.27532310	0.00000000

G1C

E: −3838.35
H: −3707.78
G: −3743.98
N_{imag} = 0

H	-4.53871030	-2.83945566	0.00000000

[S15] C. Y. Legault, *CYLview20*, Université de Sherbrooke, Sherbrooke, Quebec, Canada, **2020**, www.cylview.org.

N	3.51294227	0.61127058	0.00000000
N	−0.03713328	2.56836403	0.00000000
O	−2.41544039	1.16142947	0.00000000
H	−4.66726530	−0.34440221	0.00000000
C	3.54450633	1.97622068	0.00000000
H	2.39239442	3.77145087	0.00000000
H	−0.06598931	3.57879410	0.00000000
H	−0.16177738	−3.88060280	0.00000000
H	−0.43193297	−0.35984702	0.00000000
C	−2.50874849	−0.09167037	0.00000000
N	−2.47322807	−2.95839605	0.00000000
C	1.14796652	1.94007972	0.00000000
H	0.74521637	−2.36106294	0.00000000
H	4.36532309	0.06038520	0.00000000
C	−3.71508311	−0.86339785	0.00000000
C	2.38477202	2.68709509	0.00000000
H	4.52599223	2.44108875	0.00000000
H	−0.93609925	2.02710193	0.00000000
N	1.14049486	0.59361407	0.00000000
C	−3.62733895	−2.24015827	0.00000000
N	−1.34037728	−0.87259284	0.00000000
N	−0.14709599	−2.87013057	0.00000000
C	−1.34614945	−2.24417683	0.00000000
C	2.29674187	−0.12529981	0.00000000
O	2.35601954	−1.36570130	0.00000000

G2C

E: −3834.20
H: −3703.82
G: −3740.00
N_{imag} = 0

H	−4.72704803	−2.49623084	0.00000000
N	3.43245389	0.76608665	0.00000000
N	−0.14432090	2.67303698	0.00000000
O	−2.47743331	1.23797285	0.00000000
H	−0.18893100	3.68310642	0.00000000
C	3.44540725	2.13207040	0.00000000
H	2.26990894	3.91157020	0.00000000
H	−2.65865171	−3.88793136	0.00000000
H	−0.11673060	−3.75443531	0.00000000
H	−0.50096617	−0.24858433	0.00000000
C	−2.58788387	−0.00942404	0.00000000
C	−2.60364026	−2.80448922	0.00000000
C	1.04889720	2.06449515	0.00000000
H	0.72423486	−2.21097827	0.00000000
H	4.29223439	0.22697092	0.00000000
N	−3.78446533	−0.66902595	0.00000000
C	2.27652075	2.82719813	0.00000000
H	4.42065519	2.60978826	0.00000000
H	−1.03691765	2.11441737	0.00000000
N	1.06166730	0.71649310	0.00000000
C	−3.75100787	−2.00488232	0.00000000
N	−1.39815732	−0.77924748	0.00000000
N	−0.15728132	−2.74566566	0.00000000
C	−1.36746469	−2.14145199	0.00000000
C	2.22630901	0.01539186	0.00000000
O	2.30261127	−1.22625152	0.00000000

G3C

E: −3825.10
H: −3694.98
G: −3731.42
N_{imag} = 0

O	−2.40332505	1.14095745	−0.01218692
H	−4.64459113	−0.35568229	−0.06050698
H	−0.08056830	−3.89130450	0.05776842
H	−0.41199042	−0.37332200	0.04955531
C	−2.48494082	−0.11626463	0.00180208
C	−2.55451851	−2.90804147	0.03074458
H	0.77965772	−2.36467112	0.06964309
C	−3.69768375	−0.89057514	−0.02580208
N	−3.71046331	−2.21693726	−0.01320901
N	−1.31873250	−0.89227291	0.04611741
N	−0.10597814	−2.88472778	0.13333950
C	−1.31129046	−2.25949799	0.06446419
H	−2.60589802	−3.99491477	0.04310888
O	2.37055206	−1.36510921	−0.00007243
N	3.51697393	0.61778893	−0.00761645
N	−0.04320142	2.55642598	0.01494704
C	3.54133871	1.98317552	−0.00831348
H	2.38018953	3.77231887	−0.00148537
H	−0.07675603	3.56682798	0.00920301
C	1.14463054	1.93530075	0.00931454
H	4.37219299	0.07140801	−0.01457486
C	2.37810002	2.68794766	−0.00009732
H	4.52038701	2.45302263	−0.01619952
H	−0.94144059	2.00980091	0.00953113
N	1.14520549	0.58785763	0.01279052
C	2.30485161	−0.12420288	0.00200518

***C_s* symmetry enforced**
E: −3825.10
H: −3695.59
G: −3730.38
N_{imag} = 1 (−27.8594 cm^{-1})

O	−2.40347566	1.14348257	0.00000000
H	−4.64594696	−0.35252231	0.00000000
H	−0.07999616	−3.89211259	0.00000000
H	−0.41185261	−0.37137865	0.00000000
C	−2.48538321	−0.11388775	0.00000000
C	−2.55506554	−2.90587663	0.00000000
H	0.78263014	−2.36287305	0.00000000
C	−3.69855757	−0.88765097	0.00000000
N	−3.71126398	−2.21421910	0.00000000
N	−1.31853950	−0.89027251	0.00000000
N	−0.10496639	−2.88309737	0.00000000
C	−1.31079766	−2.25770403	0.00000000
H	−2.60641097	−3.99283687	0.00000000
O	2.37213181	−1.36278821	0.00000000
N	3.51782577	0.62049921	0.00000000
N	−0.04307029	2.55794573	0.00000000
C	3.54172410	1.98591428	0.00000000
H	2.37996269	3.77465523	0.00000000
H	−0.07684718	3.56836221	0.00000000
C	1.14494336	1.93723141	0.00000000
H	4.37326083	0.07441367	0.00000000
C	2.37822446	2.69028077	0.00000000
H	4.52064757	2.45608695	0.00000000
H	−0.94137339	2.01117547	0.00000000
N	1.14601563	0.58974958	0.00000000
C	2.30592422	−0.12183003	0.00000000

GC1

E: −4388.20
H: −4246.82
G: −4284.46

N_{imag} = 0

N	-4.25485308	-2.46326029	0.00000000
C	4.22543327	0.86421313	0.00000000
N	0.60227873	2.80027217	0.00000000
O	-1.75496943	1.43047296	0.00000000
N	-4.41957388	-0.21319516	0.00000000
C	4.17288027	2.21404500	0.00000000
H	2.91803979	3.87590548	0.00000000
H	0.58064784	3.81072294	0.00000000
H	0.50070613	-3.63104182	0.00000000
H	0.18674758	-0.13767797	0.00000000
C	-1.87794365	0.18513681	0.00000000
N	-1.83561907	-2.75135646	0.00000000
C	1.76768024	2.13132830	0.00000000
H	1.37768215	-2.09293554	0.00000000
H	5.17079113	0.33349217	0.00000000
C	-3.07851073	-0.59300343	0.00000000
N	2.94924583	2.86334587	0.00000000
H	5.04585044	2.85940131	0.00000000
H	-0.30424424	2.26394106	0.00000000
N	1.78174223	0.80829103	0.00000000
C	-2.95508468	-1.99698509	0.00000000
N	-0.72704742	-0.63773655	0.00000000
N	0.49191568	-2.62081980	0.00000000
C	-0.71986461	-2.01898718	0.00000000
C	2.97542930	0.09395280	0.00000000
O	2.96705871	-1.15296360	0.00000000
C	-5.09073838	-1.34332381	0.00000000
H	-6.16934189	-1.44160929	0.00000000
H	-4.52633824	-3.43962505	0.00000000

G1C1

E: -3830.07
H: -3699.90
G: -3736.53
N_{imag} = 0

H	-4.55447672	-2.79633147	0.00000000
C	3.59538651	0.61520537	0.00000000
N	-0.02958438	2.55118837	0.00000000
O	-2.38970179	1.18224713	0.00000000
H	-4.65596544	-0.29976049	0.00000000
C	3.54261595	1.96515203	0.00000000
H	2.28923849	3.62844178	0.00000000
H	-0.05126361	3.56169791	0.00000000
H	-0.18340555	-3.87967725	0.00000000
H	-0.42056345	-0.36340973	0.00000000
C	-2.49498542	-0.07263038	0.00000000
N	-2.49110344	-2.93799860	0.00000000
C	1.13746646	1.88457283	0.00000000
H	0.73560803	-2.36261375	0.00000000
H	4.54104393	0.08498100	0.00000000
C	-3.70944128	-0.82891144	0.00000000
N	2.31924951	2.61583959	0.00000000
H	4.41586280	2.61012939	0.00000000
H	-0.93788089	2.01316080	0.00000000
N	1.15320887	0.56189889	0.00000000
C	-3.63625405	-2.20750706	0.00000000
N	-1.33674395	-0.86365749	0.00000000
N	-0.16205992	-2.86920658	0.00000000
C	-1.35521543	-2.23591219	0.00000000
C	2.34537711	-0.15517005	0.00000000
O	2.33358766	-1.40172862	0.00000000

G2C1

E: −3826.18
H: −3696.21
G: −3732.73
N_{imag} = 0

H	−4.73919204	−2.45029315	0.00000000
C	3.51641124	0.77174454	0.00000000
N	−0.13512214	2.65542170	0.00000000
O	−2.45159096	1.26031786	0.00000000
H	−0.17271347	3.66569144	0.00000000
C	3.44503080	2.12115684	0.00000000
H	2.16993586	3.76734311	0.00000000
H	−2.68648612	−3.86442496	0.00000000
H	−0.13740703	−3.75240809	0.00000000
H	−0.48864428	−0.25053873	0.00000000
C	−2.57344688	0.01098378	0.00000000
C	−2.61978034	−2.78163693	0.00000000
C	1.04073019	2.00895331	0.00000000
H	0.71674677	−2.21086306	0.00000000
H	4.46916213	0.25443545	0.00000000
N	−3.77660724	−0.63281142	0.00000000
N	2.21355185	2.75518802	0.00000000
H	4.30967502	2.77761485	0.00000000
H	−1.03773593	2.09910739	0.00000000
N	1.07663540	0.68499465	0.00000000
C	−3.75768521	−1.97022221	0.00000000
N	−1.39379421	−0.76868494	0.00000000
N	−0.17085126	−2.74322017	0.00000000
C	−1.37543289	−2.13192526	0.00000000
C	2.27646462	−0.01356029	0.00000000
O	2.28214614	−1.26236374	0.00000000

G3C1

E: −3816.95
H: −3687.85
G: −3722.97
N_{imag} = 0

O	−4.89773766	−3.46947648	0.00000000
H	−7.58919533	−3.32988117	0.00000000
H	−5.46977121	2.04541243	0.00000000
H	−3.96156133	−1.14525070	0.00000000
C	−5.61205096	−2.42796864	0.00000000
C	−7.09968576	−0.06626180	0.00000000
H	−3.94565900	1.16472719	0.00000000
C	−7.04855319	−2.38573914	0.00000000
N	−7.73839664	−1.25128984	0.00000000
N	−5.00674630	−1.16656602	0.00000000
N	−4.97813343	1.16379392	0.00000000
C	−5.69775435	0.01390120	0.00000000
H	−7.69986918	0.84146299	0.00000000
O	−2.09791718	1.12185655	0.00000000
C	0.00000000	0.00000000	0.00000000
N	−2.18153281	−3.48267130	0.00000000
C	0.62579249	−1.19742495	0.00000000
H	0.36639110	−3.26364665	0.00000000
H	−1.69698857	−4.36985846	0.00000000
C	−1.50059807	−2.32521811	0.00000000
H	0.55637134	0.93048138	0.00000000
N	−0.11146667	−2.37034411	0.00000000
H	1.70418747	−1.32228419	0.00000000
H	−3.24006901	−3.46841624	0.00000000

N	−2.14452624	−1.16854602	0.00000000
C	−1.46720535	0.04547822	0.00000000

G

E: −2462.27
H: −2385.97
G: −2412.78
N_{imag} = 0

N	0.28163300	−2.55390325	0.20817153
O	0.76823470	2.07859287	−0.01768081
N	0.63775132	−0.23020992	0.06076946
C	1.16821770	−1.49882350	0.09458302
N	2.46495165	−1.74127190	0.07448973
C	3.21532061	−0.60753286	0.03420043
C	2.79461981	0.73190991	−0.01188713
C	1.38215917	1.02021631	0.00312451
N	4.59273312	−0.54920346	0.01590087
C	4.94045783	0.80057138	−0.03825888
N	3.88787244	1.58983610	−0.05606015
H	−0.63607368	−2.44724072	−0.21002029
H	0.71157694	−3.46001864	0.05244828
H	−0.36822411	−0.09582809	0.13341145
H	5.21493797	−1.34918163	0.04019435
H	5.97483178	1.12005880	−0.06263857

G1

E: −1903.95
H: −1839.48
G: −1863.58
N_{imag} = 0

H	−4.52117811	−2.84677250	−0.00171088
H	0.70733971	−2.43139339	−0.13024692
C	−1.33819444	−2.23958897	0.10180237
O	−2.40727582	1.16418976	−0.02751152
H	−4.67047811	−0.36037867	−0.08392562
C	−3.71397377	−0.86929596	−0.03311799
C	−3.61871886	−2.23622409	0.01322023
N	−1.33111303	−0.87032306	0.06858322
H	−0.17928316	−3.88717247	0.07087873
H	−0.46246332	−0.34317967	0.13653020
C	−2.51744378	−0.06075038	−0.00412612
N	−2.44885369	−2.94936887	0.07091266
N	−0.12777842	−2.88513639	0.22087350

G2

E: −1897.66
H: −1833.43
G: −1857.75
N_{imag} = 0

H	−4.71367530	−2.50406997	−0.04641725
H	0.68659341	−2.25487255	−0.10898152
C	−1.36386587	−2.13480577	0.07596088
O	−2.47478712	1.24464764	−0.02607231
N	−3.79186856	−0.67534098	−0.04659067
C	−3.74477505	−1.99883958	−0.01756782
N	−1.39365518	−0.77470162	0.04497436
H	−2.63619449	−3.88131149	0.05954419
H	−0.12066792	−3.75339800	0.00809334
H	−0.53659649	−0.22903903	0.09952333
C	−2.60433181	0.02723696	−0.01246009
C	−2.58408710	−2.79839840	0.04116500

N	−0.14237397	−2.75693655	0.18846532

G3
E: −1890.00
H: −1825.88
G: −1850.08
N_{imag} = 0

N	−0.07219144	−2.87606742	0.24283577
H	0.72657041	−2.42300330	−0.19210576
C	−1.30141660	−2.24889230	0.09960153
O	−2.42155052	1.14336591	−0.00961628
H	−4.65640197	−0.38652402	−0.09757511
C	−3.70302264	−0.90980293	−0.04395109
N	−3.69693128	−2.22394737	−0.02022879
N	−1.31553484	−0.87953096	0.07736210
H	−0.08471515	−3.87841550	0.08537554
H	−0.45083718	−0.34823751	0.16410049
C	−2.50026322	−0.08606147	0.00268588
C	−2.52071959	−2.90559678	0.04273240
H	−2.56265731	−3.99193400	0.05094517

C
E: −1904.41
H: −1839.96
G: −1864.39
N_{imag} = 0

O	1.71216494	−0.12514094	−2.04497528
H	2.31218647	−0.10124492	0.34154551
N	1.31941154	0.06340310	0.20760262
N	−0.46679176	0.28255734	−1.36453488
C	0.87744261	0.06603261	−1.16715763
C	−1.27528857	0.48247398	−0.33179909
C	−0.84261719	0.48248795	1.04339355
C	0.48572159	0.26342989	1.26125152
N	−2.59141927	0.72372102	−0.61541491
H	−1.52798514	0.65282284	1.86625504
H	0.92805887	0.24038909	2.25353360
H	−2.88830433	0.60957048	−1.57727041
H	−3.29010532	0.71235125	0.11368437

C1
E: −1894.20
H: −1829.80
G: −1854.16
N_{imag} = 0

C	2.96165546	0.09337808	0.01905012
C	4.21645371	0.87166236	−0.04998703
N	0.62717982	2.87455655	0.23214548
H	5.15525674	0.33207908	−0.11602454
O	2.94686720	−1.13685307	0.02626049
C	4.18722380	2.22038127	−0.01350311
H	2.94446599	3.88249408	0.21958812
H	0.57769380	3.76578312	−0.25251911
N	2.96831821	2.88212371	0.06193646
H	5.06697350	2.85625947	−0.04139434
H	−0.20076144	2.29728267	0.11459977
N	1.75828482	0.84118812	0.06405339
C	1.79717957	2.13907897	0.09962044
